# Salinity-induced physiological, antioxidative, and enzymatic responses of ‘Oliana’ and ‘Lecciana’ olive tree cultivars

**DOI:** 10.3389/fpls.2025.1746013

**Published:** 2026-01-28

**Authors:** Khalid Hussain, John-Paul Fox, Lukas M. Hallman, Muhammad Adnan Shahid, Lorenzo Rossi

**Affiliations:** 1 Department of Horticultural Sciences, Texas A&M University, College Station, TX, United States; 2 Indian River Research and Education Center, Horticultural Sciences Department, Institute of Food and Agricultural Sciences, University of Florida, Fort Pierce, FL, United States; 3 North Florida Research and Education Center, Horticultural Sciences Department, Institute of Food and Agricultural Sciences, University of Florida, Quincy, FL, United States

**Keywords:** antioxidant defense, fluorescence, *Olea europaea*, oxidative stress, salinity tolerance

## Abstract

As land salinization intensifies across the United States, there is growing interest in cultivating salt-tolerant crops such as olives (*Olea europaea*). With extra virgin olive oil becoming an increasingly popular and valuable commodity in the U.S. market, growing olive cultivars that can thrive under challenging conditions is critical. This study evaluated the physiological, antioxidative and biochemical responses of two relatively newly introduced olive cultivars, ‘Oliana’ and ‘Lecciana’, to salinity stress under controlled greenhouse conditions. Eight-month-old plants were subjected to three salinity treatments (0, 50, and 100 mM NaCl) in a completely randomized design (*n* = 9). Plant gas exchange parameters were measured at 0, 15, 30, and 45 days after treatment, while chlorophyll content, fluorescence (Fv/Fm), biomass, nutrient accumulation (in leaves, stems, and roots), antioxidant enzyme activity, osmolyte levels, and reactive oxygen species (ROS) concentrations were assessed at 15, 30, and 45 days. Salinity stress significantly reduced gas exchange parameters in ‘Lecciana’ cultivar as compared to ‘Oliana’, resulting in decreased chlorophylls, Fv/Fm, and nutrient content. In response, antioxidant enzymes and osmolyte accumulation (proline and glycine betaine) increased with ‘Lecciana’ showing the strongest response. Conversely, lipid peroxidation and hydrogen peroxide (H_2_O_2_) levels were highest in ‘Oliana’ under 100 mM NaCl, indicating greater oxidative stress. These findings suggest that ‘Lecciana’s high salinity tolerance is associated with enhanced antioxidant defense and compatible solutes. In addition, ‘Lecciana’ and ‘Oliana’ could be a promising cultivar for saline and drought-prone regions of the U.S., supporting the expanding production of high-quality extra virgin olive oil.

## 1 Introduction

In recent years, the demand for olive oil, specifically the Extra Virgin Olive Oil (EVOO), is continuously increasing in the United States due to its potential health benefits *i.e.*, monounsaturated fats, antioxidants, and anti-inflammatory properties (Ghanbari et al., 2012), making it a preferred alternative to other cooking oils. California has emerged as the central hub for olive oil production in the United States due to its favorable Mediterranean-like climate. The state produces 99% of the olives in the United States, encompassing over 17,401 hectares of bearing trees and producing approximately 162,500 Tons, valued at around $144 million (USDA 2024). In addition to California, other states including Texas, Arizona, Georgia, and Florida have begun producing olives, however, this only accounts for about 3% of total olive oil production in the United States (Hwang and Quadri-Felitti, 2022), highlighting the need to expand the national production by cultivating new varieties. Additionally, drought is a significant stressor in California, requiring crops to be irrigated; however, irrigation water is often of lower quality with higher salinity, and its use under water-limited conditions contributes to land salinization in major agricultural regions such as the San Joaquin Valley (Letey, 2000; Schoups et al., 2005; Helalia et al., 2021).

As soil salinization increases across the US, it is critical to grow salt-tolerant cultivars as salinity stress remains a major challenge due to high evapotranspiration and insufficient leaching of ions. This leads to salt accumulation in the root zone (Gucci and Tattini, 2010), which has detrimental impacts on olive plant growth (Rossi et al., 2016a; Wang and Huang, 2019). Although, olive trees have moderate tolerance to salt stress, this trait is cultivar-dependent (Chartzoulakis, 2005; Tabatabaei, 2007; Tattini and Traversi, 2009) with ‘Leccino’ classified as salt sensitive, whereas ‘Frantoio’ demonstrates greater tolerance (Rossi et al., 2016a).

To cope with salt stress, plants modify their morphology, physiology, and anatomy (Rossi et al., 2015, 2016, 2017; Adams et al., 2019; Shahid et al., 2020; Li et al., 2024; Hussain et al., 2025) as salt stress disrupts the various cellular processes (Bazakos et al., 2012). Understanding the physiological processes that regulate crop growth under salinity stress is essential to provide significant insights for improving crop yield (Gupta et al., 2020). Salinity induced stress effects on leaf stomatal conductance and photosynthesis are influenced by duration, and specific plant species (Liu et al., 2017). For example, Loreto et al. (2003) observed that salinity stress inhibits the photosynthetic rate in olive plants primarily due to the stomatal closure, as higher salt stress alter the structure of stomata and chloroplasts and impairs the chlorophyll pigment that ultimately affects the photosynthetic rate (Arif et al., 2020). Short exposure of salt stress causes chlorophyll degradation which escalate to severe levels with prolonged stress (Hossain et al., 2017). It induces osmotic and ionic stress, as well as nutrient imbalances, that can lead to multiple disruptions to plant physiological processes which leads to excessive reactive oxygen species (ROS) formation (Arif et al., 2020). However, in woody perennials, these mechanisms differ due to their long lifespan and structural complexity, including compartmentalization of ions in older tissues, specific proteins synthesis, production of compatible solutes and maintenance of hydraulic function across multiple growing seasons (Chen and Polle, 2010; Llanes et al., 2021). Loupassaki et al. (2002) reported that uptake of N, K, Ca, and Mg decreased in six Greek olive cultivars under salt stress in root tissue while P was not affected, however, in young leaves N, P, Ca and Mg dropped.

Nevertheless, plants have the capacity to develop tolerance to environmental stresses through the synthesis of antioxidative enzymes (Hasanuzzaman et al., 2020; Garcia-Caparros et al., 2021); which can mitigate the negative effects of oxidative stress by detoxifying the excessive ROS (Sachdev et al., 2021). The major components of this defense system are superoxide dismutase (SOD), catalase (CAT), ascorbate peroxidase (APX), guaiacol peroxidase (GPX), monodehydroascorbate reductase (MDAR), dehydroascorbate reductase (DHAR), and glutathione reductase (GR) (Hasanuzzaman et al., 2020; Wang et al., 2024). For instance, Goreta et al. (2007) reported that olive genotypes accumulated higher SOD concentration suggesting it may act as a precursor to the earlier onset of oxidative stress. Additionally, Sofo et al. (2005) observed enhanced activities of CAT, APX and GPX in ‘Coratina’ olive cultivar under drought stress. Moreover, various osmolytes enhance salt tolerance (Hussain et al., 2024) and among them proline is an important signaling molecule that accumulates in cytosol and protects membranes; in addition, it also lowers the buildup of harmful ions under salt stress (Zhang and Dai, 2019; Hosseinifard et al., 2022). Similarly, Bashir et al. (2021) reported that proline accumulation in the ‘Canino’ olive cultivar was 36% higher as compared to salt sensitive ‘Sirole’ cultivar under salt stress. Moreover, GB accumulates in the plant cells and maintains osmotic balance by regulating the Na^+^ to K^+^ ratio, which reduces the effects of ion toxicity (Singh et al., 2015).

Both ‘Oliana’ and ‘Lecciana’ are being introduced due to their suitability to high-density plantings and ability to produce high quality olive oil (Camposeo et al., 2021; Lodolini et al., 2023) however, their suitability to saline soils is understudied. Moreover, differing parentage of ‘Oliana’ (‘Arbequina’ × ‘Arbosana’) and ‘Lecciana’ (‘Arbosana’ × ‘Leccino’) indicate that they may exhibit different physiological and biochemical strategies when exposed to salt stress. For example, comparative physiological data showed differential salinity stress responses among cultivars including ‘Oliana’ and ‘Lecciana’ (Palm et al., 2024). Similarly, Hussain et al. (2025) reported that both these cultivars showed distinct root morphological and anatomical responses under salt stress highlighting cultivar specific mechanisms of salinity tolerance; however, studies on antioxidative and enzymatic responses remain limited, restricting a comprehensive mechanistic understanding of these cultivars. Therefore, this detailed study was designed to determine their tolerance level to varying salinity stress by examining their growth, physiological and antioxidative and enzymatic traits.

## 2 Materials and methods

### 2.1 Plant material and growth conditions

This greenhouse study was conducted at the University of Florida, Institute of Food and Agricultural Sciences (UF/IFAS), Indian River Research and Education Center (IRREC), Fort Pierce, FL, USA (Lat. 27°25’34.2” N; Long. –80°24’34.0” W). Mean day/night temperatures were 37°C/27°C, with relative humidity of 85%/75% respectively. Eight-month-old plants of ‘Oliana’ and ‘Lecciana’ olive (*Olea europaea*) cultivars obtained from Agromillora nursery located in Gridley, California (USA) were thoroughly washed to remove residual nursery substrate and potted in 3 L containers with a 3:1 mix of play sand (Atlanta, GA, USA) and clay pebbles (Petaluma, CA, USA). The plants were irrigated with Hoagland solution (half strength) obtained from Phyto Technology Lab, Shawnee Mission, KS (USA) during a 45-day establishment phase (Hoagland and Arnon, 1938). Plants were then subjected to three different salinity levels including 0 mM NaCl (control), 50 mM NaCl, and 100 mM NaCl. Physiological responses were recorded at 0, 15, 30, and 45 days after treatment (DAT), while plants were harvested at three different time points for analyses of biomass, mineral nutrients, antioxidant enzyme activity, and osmolyte concentrations.

### 2.2 Plant biomass and physiological traits measurements

The number of lateral shoots were counted while their length was measured using a ruler. Plant physiological parameters (stomatal conductance, transpiration rate, intercellular CO_2_, and CO_2_ assimilation rate) were recorded using a Li-6800 (Li-Cor, Lincoln, NE, USA). Plants were harvested at 45 DAT, and roots were rinsed twice with distilled water. The shoots and roots were separated to measure the fresh weight (g) of above- and below-ground tissues and measurements were taken using a digital balance (Sartorius, Göttingen, Germany).

### 2.3 Chlorophyll contents measurements

For Chl quantification, samples of 100 mg leaf lamina per treatment were weighed precisely and then placed into 25 mL of N, N-dimethylformamide (DMF) in individual tubes, and kept at 4°C in the dark over a period of 48 hours. Following this period, absorbance was measured at 664 and 647 nm in 3.5 mL quartz cuvettes using a Thermo Scientific GENESYS 50 UV-Visible Spectrophotometer (Hampton, NH, USA). Concentrations of Chl a, Chl b, and total Chl were calculated following the formulas of Inskeep and Bloom (1985):


$$\mathrm{Chl}\;a=12.70\times {\text{A}}_{664}–2.79\times {\text{A}}_{647}$$

$$\mathrm{Chl}\;b=20.70\times {\text{A}}_{647}–4.62\times {\text{A}}_{664}$$

$$\mathrm{Total}\;\mathrm{Chl}=17.90\times {\text{A}}_{647}+8.08\times {\text{A}}_{664}$$

### 2.4 Fv/Fm measurements

Chl fluorescence (F_v_/F_m_) was measured at 15-day intervals using a modulated fluorometer (OS30p+, Opti-Sciences, Hudson, NH, USA). Leaves were dark-acclimated in clips for at least 30 min prior to fluorescence recording, and the F_v_/F_m_ was determined immediately following a 1-s exposure to red light (660 nm).

### 2.5 Mineral contents of leaf, stem and root tissues

Plants were harvested at three different time intervals (15, 30, and 45 DAT), separated into root, stem, and leaf and oven dried. For mineral analysis, 0.5 g of each dried sample was ground in a Thomas Wiley mill (Thomas Scientific, Swedesboro, NJ, USA) and then sieved through a 1.0 mm mesh and transferred to 50 mL vials. Subsequently, 5 mL of HNO_3_ was added, and the volume was adjusted to 20 mL and placed at 95°C for 90 min on a DigiBlock 3000 digestor (SPC Science, USA). Following this, 4 mL of 30% H_2_O_2_ was added, and digestion proceeded for an additional 20 min. After that, samples were cooled and their volume adjusted to 50 mL with distilled water. Nutrient analysis was performed by using inductively coupled plasma mass spectrometry (ICP-MS; Spectro Ciros CCD, Freiburg, MA, USA). The contents of N, P, Ca, Mg and S were reported as a dry mass percentage, whereas Zn was calculated in ppm.

### 2.6 Antioxidant capacity determination

Enzymatic activities were evaluated by homogenizing leaf tissue (0.5 g) in 5 mL phosphate buffer (PBS; 50 mM, pH 7.8) followed by centrifugation (15,000 × g) at 4°C for 20 min. After centrifugation, the resulting supernatant was used for enzyme assays. SOD activity was assessed via inhibition of nitroblue tetrazolium photoreduction according to Giannopolitis and Ries (1977). The CAT and POD activities were assessed by following Maehly (1954) methodology with minor modifications. For the CAT assay the reaction mixture contained PBS (50 mM, pH 7.0), H_2_O_2_ (5.9 mM), enzyme extract (0.1 mL), while POD reactions included PBS (50 mM, pH 5.0), H_2_O_2_ (40 mM), guaiacol (20 mM), and extract (0.1 mL). Absorbance was recorded every 20 s at 240 nm for CAT while at 470 nm for POD, and activities were expressed relative to total protein content.

Ascorbic acid (AsA) activity was quantified by following the protocol of Arakawa et al. (1981), and the leaf tissue (0.5 g) were homogenized in 1 mL of HClO_4_ (2.5 M) followed by centrifugation (16,000 × g) at 4°C for 20. The resulting supernatant was treated with ascorbate oxidase and absorbance was recorded at 265 nm. While glutathione (GSH) contents were determined using DTNB [5,5′-dithiobis (2-nitrobenzoic acid)] with absorbance recorded at 412 nm as outlined by Griffith (1980). The activity of ascorbate peroxidase (APX) was assessed by following the methodology of Nakano and Asada (1981) while guaiacol peroxidase (GPX) activity was recorded following the approach described by Urbanek et al. (1991). However, MDAR activity was assessed according to Arakawa et al. (1981) by monitoring the reduction in absorbance at 340 nm resulting from NADH oxidation, whereas the activity of dehydroascorbate reductase (DHAR) was measured following the methodology of Hossain and Asada (1984).

### 2.7 Stress injury parameters, oxidative damage, and osmoprotectants

Leaf tissue (0.5 g) were homogenized in a reaction mixture of PBS (0.5 mL), hydroxylammonium chloride (0.1 mL), and xanthine oxidase (1 mL), followed by incubation at 25°C for 20 min. α-Naphthylamine and sulfanilic acid (0.5 mL each) were then added, mixed, and after 20 min, absorbance was recorded at 530nm to determine superoxide levels. Hydrogen peroxide (H_2_O_2_) contents were determined by using the methodology outlined by Patterson et al. (1984). Briefly, the reaction mixture for this assay contained leaf tissue (1 g) and acetone (2 mL). The extracted supernatant was then reacted with titanium reagent, followed by the addition of ammonia solution (17 M) to form a precipitate. This precipitate was rinsed with acetone and dissolved in 2 N H_2_SO_4_ (3 mL) and its absorbance was recorded at 410 nm.

The concentrations of thiobarbituric acid (TBA) and malondialdehyde (MDA) were measured by following the methodology of Heath and Packer (1968) to determine lipid peroxidation. A mixture of equal volumes of tissue extracts and 0.5% (w/v) TBA in 20% (w/v) trichloroacetic acid was prepared, heated at 95°C for 30 min, and then rapidly cooled on ice. After centrifugation at 3,000 × g for 10 min, Absorbance readings were recorded at two different wavelengths (532 nm, 600 nm). Using a molar extinction coefficient of 155 mM^-^¹cm^-^¹, MDA levels were quantified and expressed in μmol MDA mL^-^¹ g^-^¹ DW.


MDA (nmol)=Δ (A532 nm−A600 nm)/1.56×105

Proline contents were quantified following Bates et al. (1973). Briefly, 0.5 g leaf tissue were homogenized in 10 mL solution of 3% sulfosalicylic acid, filtered, and 2 mL filtrate reacted with acid-ninhydrin and glacial acetic acid (each 2 mL) at 100°C for 1 hour. After cooling, the chromophore was extracted with 4 mL toluene, and absorbance at 520 nm was used to calculate proline (mg g^-^¹ DW). Glycine betaine (GB) levels were determined following the procedure described by Grieve and Grattan (1983).

Electrolyte leakage (EL) was measured using fresh leaf samples (stored at -80°C). Leaves were cut into uniform pieces, incubated in 10 mL of distilled water for 2–3 h at 25°C, and the initial conductivity was recorded. Samples were then placed in a water bath for 45 min at 98°C to disrupt membranes, cooled and final conductivity was measured. EL (%) was calculated as:


EL(%)=[(Initial EC/Second EC)×100]

### 2.9 Statistical analysis

The study employed a completely randomized design CRD in a factorial layout (*n* = 9). Statistical significance was assessed using a two-way Analysis of Variance (ANOVA), followed by pairwise comparisons with Tukey’s honestly significant difference (HSD) test at *p* < 0.05. Data analysis was conducted using Minitab (Minitab LLC, State College, PA, USA) and Statistix 8.1 (Analytical Software, FL, USA).

## 3 Results

### 3.1 Biomass measurements of olive tree cultivars under salt stress

**Figure 1:**
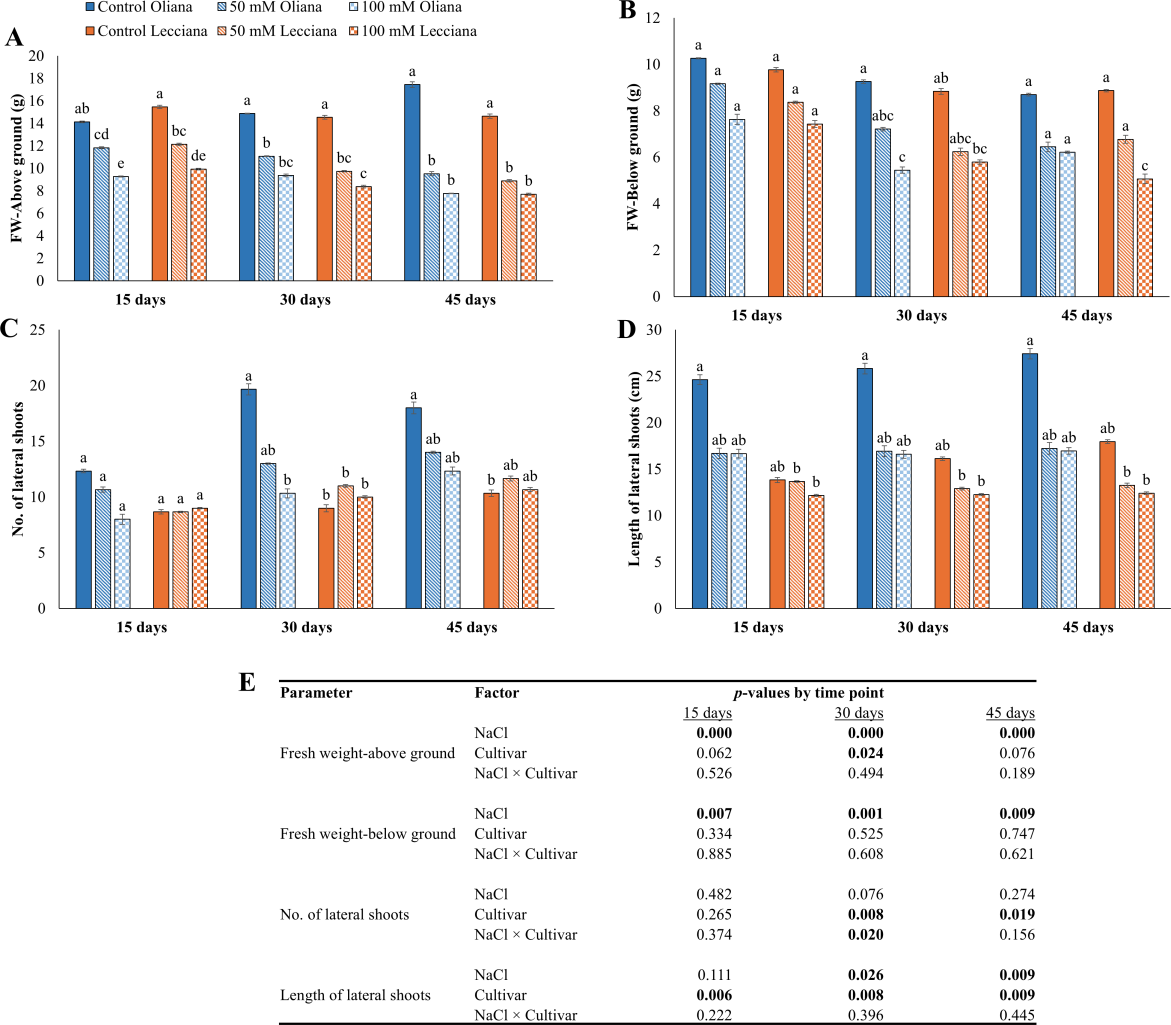
Impact of different salinity levels (0, 50, and 100 mM NaCl) on biomass and growth of ‘Oliana’ and ‘Lecciana’ olive cultivars, measured at 15, 30, and 45 days after treatment (DAT). Measurements include **(A)** above-ground fresh biomass, **(B)** below-ground fresh biomass, **(C)** number of lateral shoots, and **(D)** lateral shoot length. Significant differences (*p* < 0.05) are indicated by different letters according to Tukey’s HSD, while bars represent the standard error. Two-way ANOVA-based *p*-values for treatment, cultivar, and their interaction are shown in **(E)** with significant values in bold.

Salinity (NaCl) treated plants negatively affected the olive tree biomass in comparison to non-treated plants. A decrease in above ground fresh weight was recorded in ‘Oliana’ and ‘Lecciana’ (55% and 47% respectively). Similarly, for below ground fresh weight 28% reduction in ‘Oliana’ and 43% in ‘Lecciana’ was recorded in comparison to control. Furthermore, ANOVA findings revealed that for above and below ground fresh weight, both NaCl treatments (50 mM, 100 mM) significantly affected these traits at all three timepoints (15, 30 and 45 DAT). While, for cultivars (‘Oliana’ and ‘Lecciana’) significant differences were noted only at 30 DAT for above ground fresh weight. However, the interaction between NaCl × Cultivar was non-significant at all the three timepoints (**Figures 1A, B**).

The treatments showed no significant differences for number of lateral shoots; however, cultivar differences were significant at 30 and 45 DAT and the interaction between NaCl × Cultivar was also significant at 30 DAT only. Whereas NaCl treatments affected the length of lateral shoots at 30 and 45 DAT, and cultivars differed significantly at each time point (15, 30, and 45 DAT). The percentage reduction recorded for length of lateral shoots was 38% in ‘Oliana’ and 31% in ‘Lecciana’ cultivar (**Figures 1C, D**).

### 3.2 Chlorophyll contents of olive tree cultivars under salt stress

**Figure 2:**
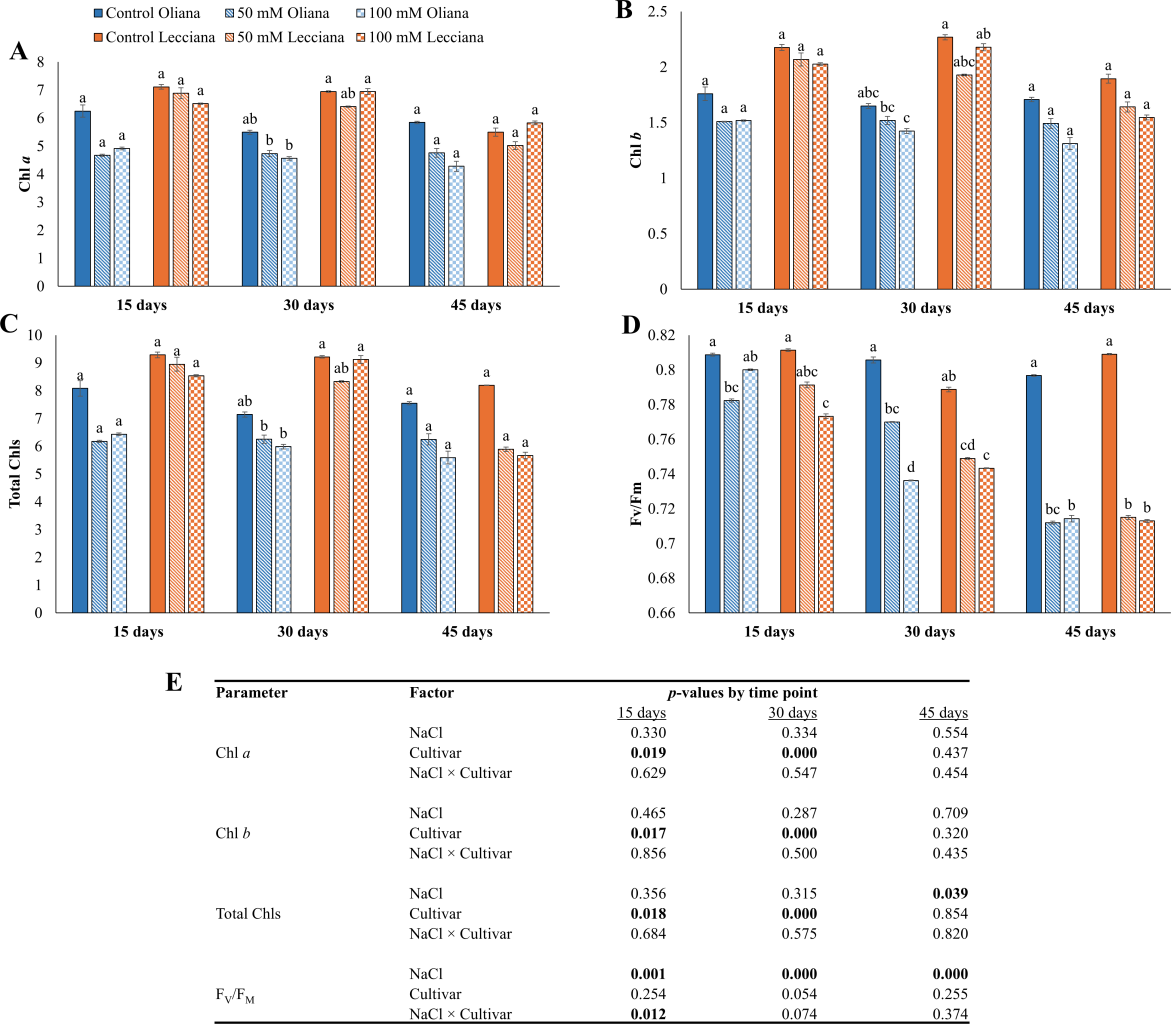
Impact of different salinity levels (0, 50, and 100 mM NaCl) on chlorophyll content and fluorescence of ‘Oliana’ and ‘Lecciana’ olive cultivars, measured at 15, 30, and 45 days after treatment (DAT). Measurements include **(A)** chlorophyll a, **(B)** chlorophyll b, **(C)** total chlorophyll, and **(D)** Fv/Fm. Significant differences (*p* < 0.05) are indicated by different letters according to Tukey’s HSD, while bars represent the standard error. Two-way ANOVA-based *p*-values for treatment, cultivar, and their interaction are shown in **(E)** with significant values in bold.

Salt treatments negatively affected the Chl contents relative to control group. In ‘Oliana’ Chl *a*, Chl *b* and total Chls decreased by 27%, 22%, and 26% respectively, while in ‘Lecciana’, only total Chls were decreased by 30%. However, ANOVA results revealed that no significant differences were recorded for the Chl *a* or *b* contents under the NaCl treatments, however both cultivars showed significant differences at 15 and 30 DAT (**Figures 2A, B**). NaCl treatments significantly affected the Chls levels at 45 DAT, however, the interaction (NaCl × Cultivar) was non-significant (**Figure 2C**).

NaCl treatments significantly affected the fluorescence (F_v_/F_m_) at all the timepoints (15, 30 and 45 DAT). A percentage decrease for F_v_/F_m_ was recorded in both cultivars ‘Oliana’ and ‘Lecciana’ (10% and 11% respectively). However, cultivars ‘Oliana’ and ‘Lecciana’ did not show any significant differences, while the interaction between NaCl × Cultivar was significant only at 30 DAT (**Figure 2D**).

### 3.3 Physiological measurements of olive tree cultivars under salt stress

**Figure 3:**
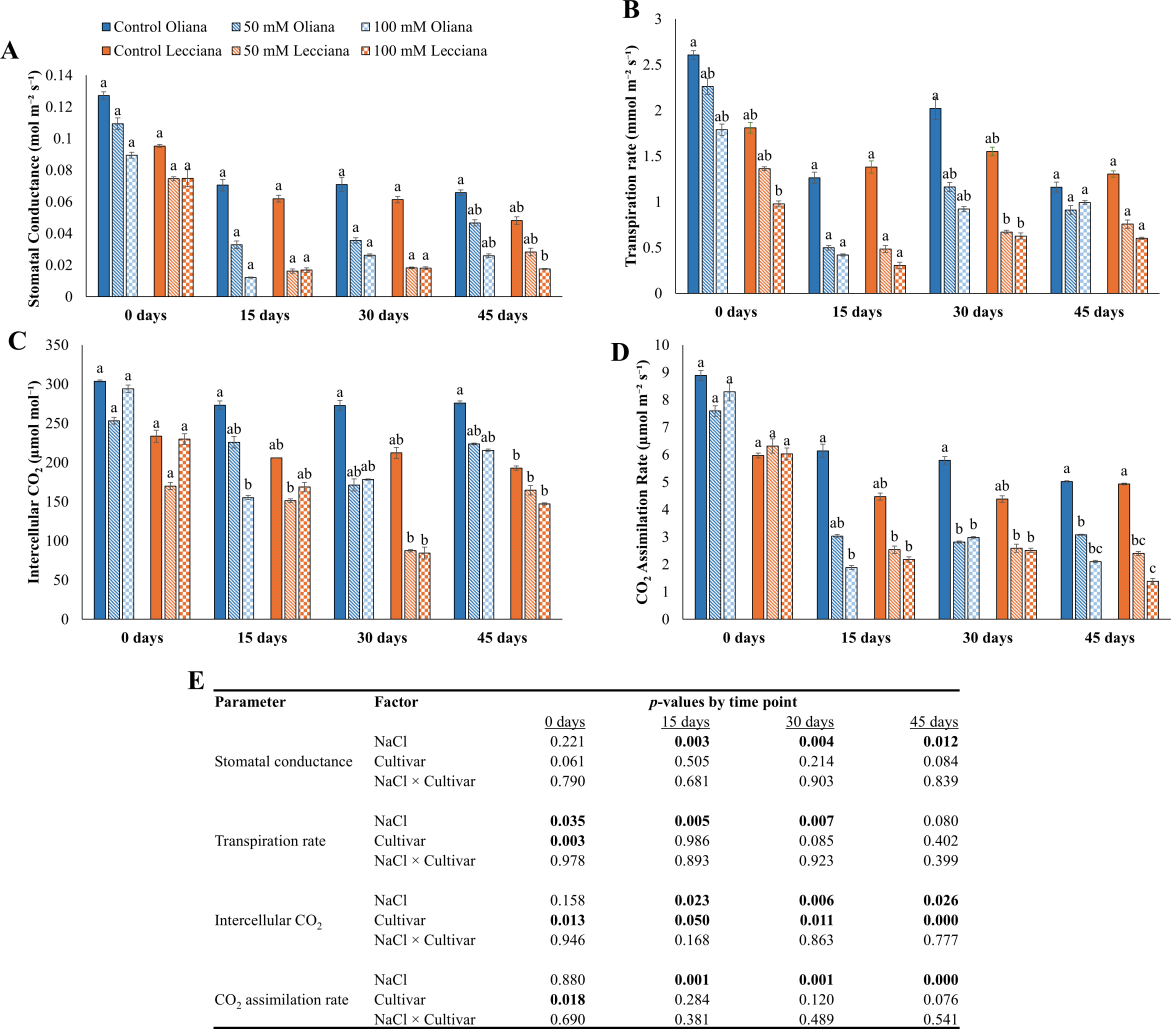
Impact of different salinity levels (0, 50, and 100 mM NaCl) on physiological parameters of ‘Oliana’ and ‘Lecciana’ olive cultivars, measured at 15, 30, and 45 days after treatment (DAT). Measurements include **(A)** stomatal conductance (mol m^-^² s^-^¹), **(B)** transpiration rate (mmol m^-^² s^-^¹), **(C)** intercellular CO_2_ (µmol mol^-^¹), and **(D)** CO_2_ assimilation rate (µmol m^-^² s^-^¹). Significant differences (*p* < 0.05) are indicated by different letters according to Tukey’s HSD, while bars represent the standard error. Two-way ANOVA-based *p*-values for treatment, cultivar, and their interaction are shown in **(E)** with significant values in bold.

Physiological parameters (stomatal conductance, transpiration rate, intercellular CO_2_, and CO_2_ assimilation rate) were significantly affected by both salt treatments (50 mM and 100 mM) (**Figure 3**). In comparison to control group, a percentage decrease was recorded in both cultivars ‘Oliana’ and ‘Lecciana’ (66% and 75% respectively) for stomatal conductance. ANOVA results revealed that NaCl treatments significantly affected the stomatal conductance at all the three timepoints (15, 30 and 45 DAT), while the cultivars and the interaction between NaCl × Cultivar was not significant (**Figures 3A, E**). Similarly, NaCl treatments negatively affected the transpiration rate as compared to control treatment and a percentage reduction of 15% and 54% in ‘Oliana’ and ‘Lecciana’ respectively. In addition, ANOVA results showed significant differences at 0, 30 and 45 DAT while cultivars also showed significant differences only at 0 DAT (**Figures 3B, E**). Similarly, intercellular CO_2_ also showed a decrease of 22% and 23% in ‘Oliana’ and ‘Lecciana’ cultivars respectively as compared to control group. The ANOVA results revealed that salinity treatments significantly impacted the intercellular CO_2_ at all the timepoints (0, 15, 30, 45 DAT). Additionally, cultivars also showed significant differences at all time points (**Figures 3C, E**). CO_2_ assimilation rate decreased in salt-treated plants relative to the control, with reductions of 58% in ‘Oliana’ and 72% in ‘Lecciana’. NaCl treatments also significantly affected the CO_2_ assimilation rate at 15, 30 and 45 DAT while the interaction (NaCl × Cultivar) was non-significant (**Figures 3D, E**).

### 3.4 Nutrient contents of olive tree cultivars under salt stress

**Figure 4:**
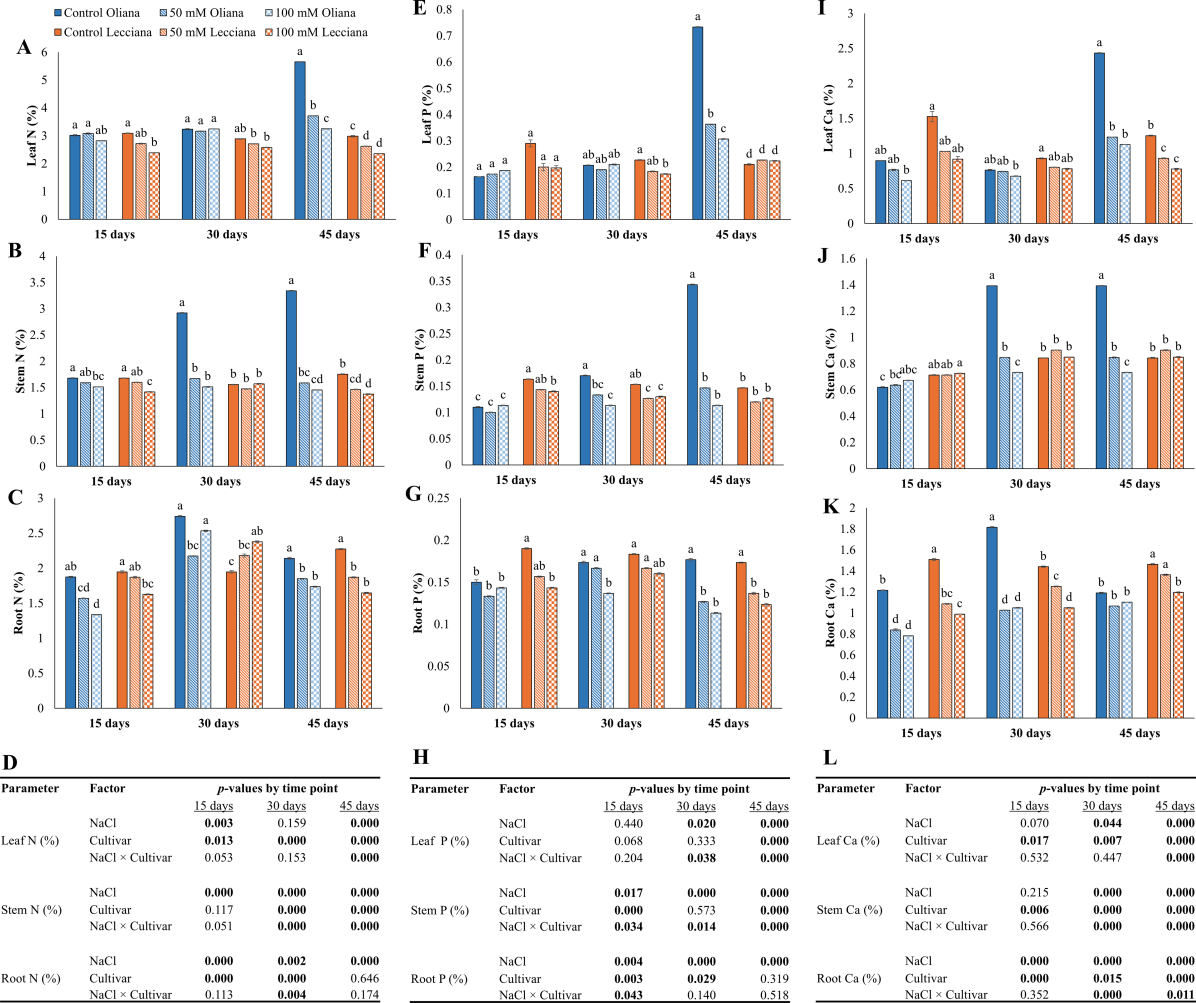
Impact of different salinity levels (0, 50, and 100 mM NaCl) on nutrient contents of ‘Oliana’ and ‘Lecciana’ olive cultivars, measured at 15, 30, and 45 days after treatment (DAT). Measurements include nitrogen (N) **(A–C)**, phosphorus (P) **(E–G)**, and calcium (Ca) **(I–K)** in leaf, stem, and root tissues. Significant differences (*p* < 0.05) are indicated by different letters according to Tukey’s HSD, while bars represent the standard error. Two-way ANOVA-based *p*-values for treatment, cultivar, and their interaction are shown in **(D, H)** and **(L)** with significant values in bold.

**Figure 5:**
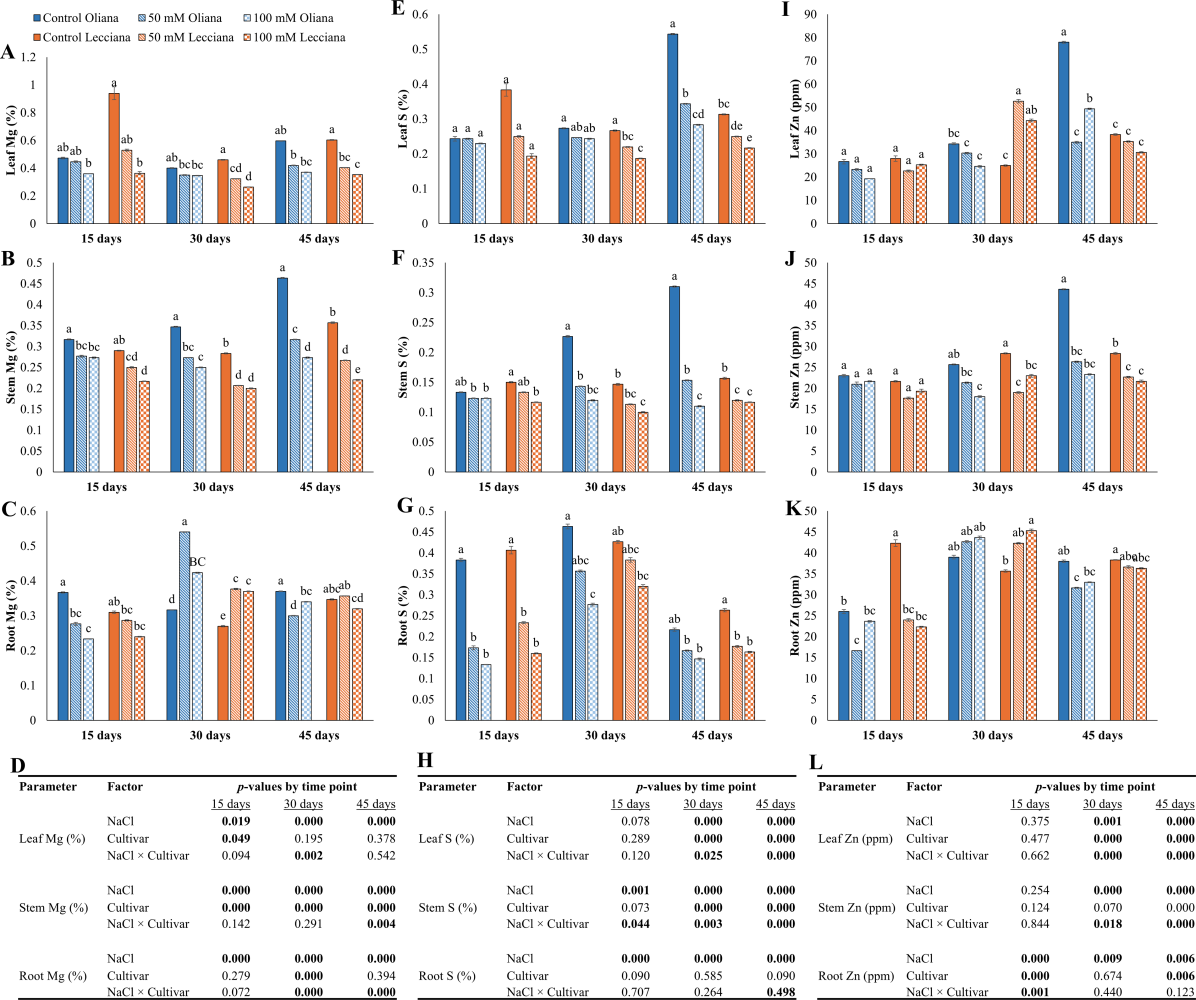
Impact of different salinity levels (0, 50, and 100 mM NaCl) on nutrient contents of ‘Oliana’ and ‘Lecciana’ olive cultivars, measured at 15, 30, and 45 days after treatment (DAT). Measurements include magnesium (Mg) **(A–C)**, sulfur (S) **(E–G)**, and zinc (Zn) **(I–K)** in leaf, stem, and root tissues. Significant differences (*p* < 0.05) are indicated by different letters according to Tukey’s HSD, while bars represent the standard error. Two-way ANOVA-based *p*-values for treatment, cultivar, and their interaction are shown in **(D, H)** and **(L)** with significant values in bold.

Both NaCl treatments negatively affected the nutrient concentrations in leaf, stem and root of olive cultivars relative to control group (**Figures 4**, **5**). ANOVA indicated that NaCl treatments significantly influenced nitrogen (N) concentrations in leaves at 15 and 45 DAT, and in stems and roots at all sampling intervals (15, 30, and 45 DAT). Additionally, the interaction between NaCl × Cultivar was significant at 45 DAT for leaf, while 30 and 45 DAT for stem and 30 DAT for root N (**Figures 4A–D**). Leaf phosphorus (P) contents were significantly impacted by NaCl treatments at 30 and 45 DAT and cultivars also showed significant differences at 45 DAT. Additionally, the NaCl × Cultivar interaction was significant at 30 and 45 DAT. Similarly, both NaCl treatments also affected the leaf and root P contents at all timepoints, while cultivars showed significant differences at 15 DAT for Leaf P contents and15 and 30 DAT for root P contents; additionally, NaCl × cultivar interaction was significant at all timepoints for leaf P while for root P contents it was significant only at 15 DAT (**Figures 4E–H**). Calcium (Ca) contents were also significantly affected by salinity stress and NaCl treatments affected the leaf and stem Ca contents at 30 and 45 DAT while in root it was affected at all the timepoints (15, 30, 45 DAT). Significant differences were recorded between the at all timepoints in leaf, stem and root while the interaction between NaCl × Cultivar was also significant at 45 DAT only for leaf Ca; 30 and 45 DAT for stem and root Ca contents (**Figures 4I–L**).

Magnesium (Mg) contents in leaf, stem and root were significantly impacted by NaCl treatments at all timepoints (15, 30, 45 DAT), while cultivars depicted significant differences at all timepoints in stem, 15 DAT in leaf and 30 DAT in root. Similarly, the interaction between NaCl × Cultivar was also significant at 30 DAT for leaf Mg, 45 DAT for stem Mg and 30 and 45 DAT for root Mg contents (**Figures 5A–D**). Sulfur (S) contents were negatively impacted by NaCl treatments and significant differences were noted at all the time-points for stem and root and for leaf at 30 and 45 DAT. Cultivars showed significant differences at 30 and 45 DAT for leaf and stem S contents and the interaction (NaCl × Cultivar) was significant at all timepoints for stem, while for leaf it was significant at 30 and 45 DAT and for root it was significant at 45 DAT (**Figures 5E–H**). Zinc (Zn) contents were also significantly impacted by NaCl treatments at all timepoints in root, while in stem and leaf at 30, 45 DAT. Cultivars ‘Oliana’ and ‘Lecciana’ showed significant differences at 30 and 45 DAT in leaf and 15 and 45 DAT in root. The interaction between NaCl × Cultivar was significant at 30 and 45 DAT in leaf and stem while in root it was significant at 15 DAT (**Figures 5I–L**).

### 3.5 Enzymatic activities of olive tree cultivars under salt stress

**Figure 6:**
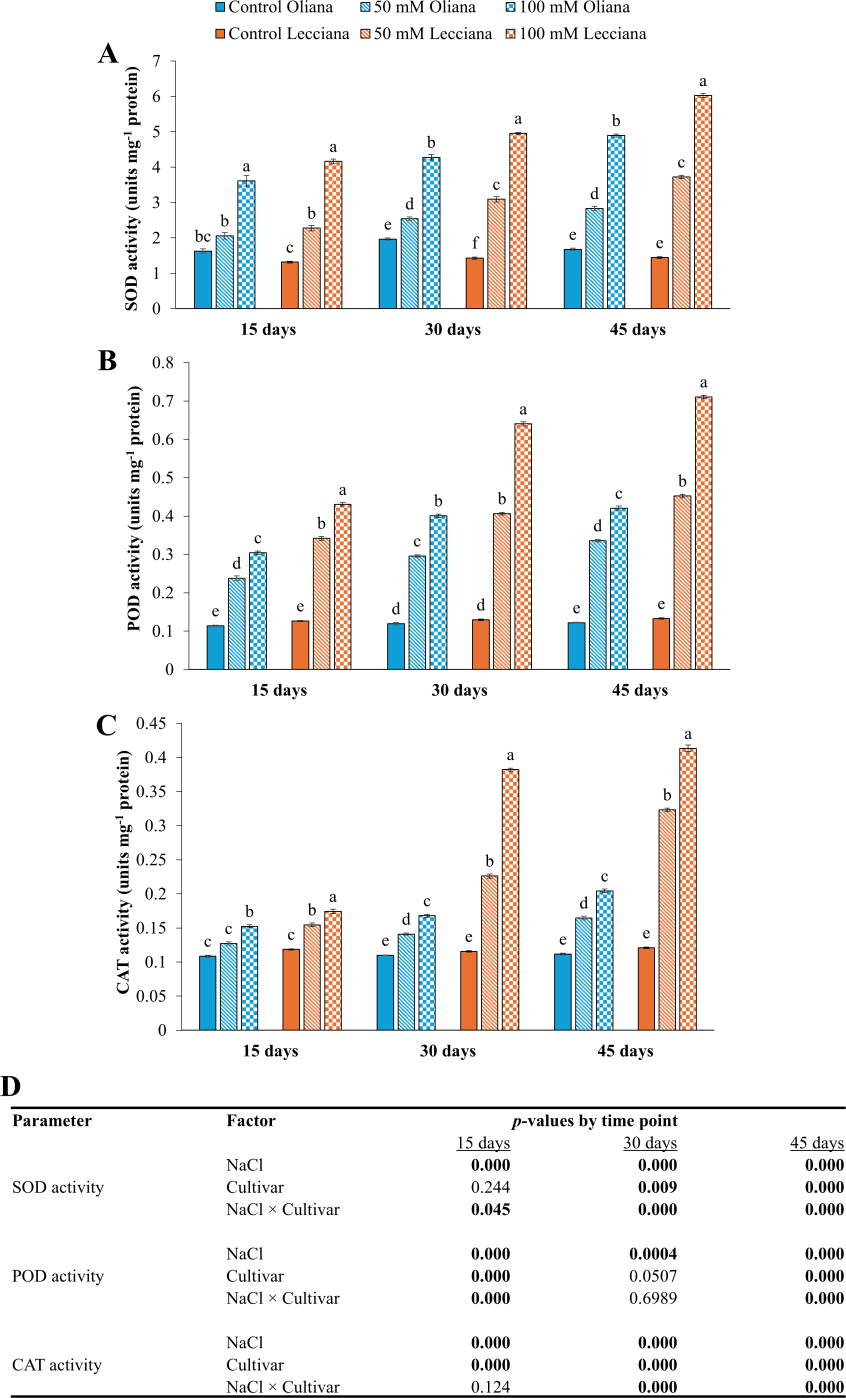
Impact of different salinity levels (0, 50, and 100 mM NaCl) on enzymatic activities of ‘Oliana’ and ‘Lecciana’ olive cultivars, measured at 15, 30, and 45 days after treatment (DAT). Measurements include **(A)** SOD activity (units mg^-^¹ protein), **(B)** POD activity (units mg^-^¹ protein), and **(C)** CAT activity (units mg^-^¹ protein). Significant differences (*p* < 0.05) are indicated by different letters according to Tukey’s HSD, while bars represent the standard error. Two-way ANOVA-based *p*-values for treatment, cultivar, and their interaction are shown in **(D)** with significant values in bold.

**Figure 7:**
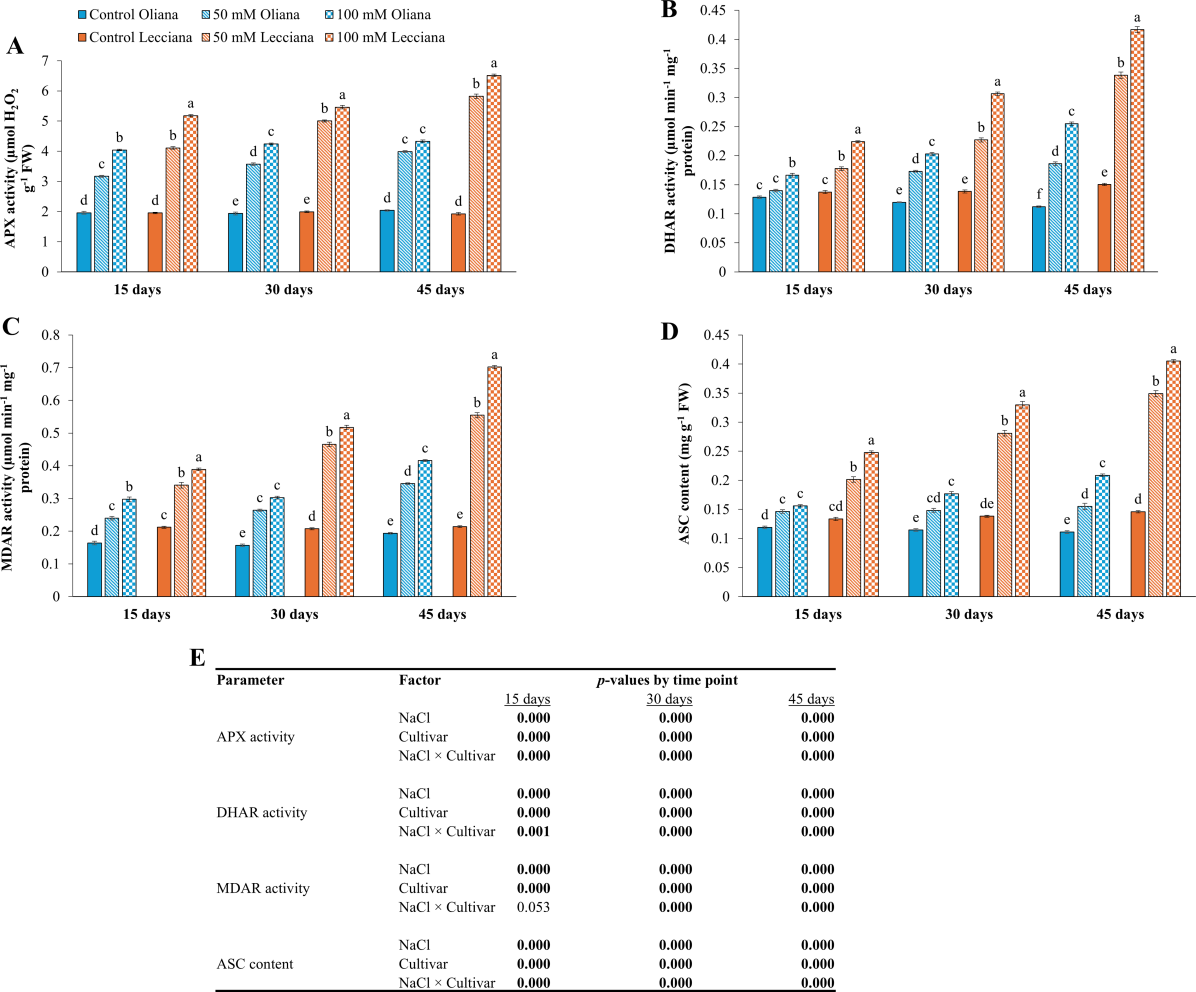
Impact of different salinity levels (0, 50, and 100 mM NaCl) on antioxidant enzyme activities and ascorbate content of ‘Oliana’ and ‘Lecciana’ olive cultivars, measured at 15, 30, and 45 days after treatment (DAT). Measurements include **(A)** APX activity (µmol H_2_O_2_ g^-^¹ FW), **(B)** DHAR activity (µmol min^-^¹ mg^-^¹ protein), **(C)** MDAR activity (µmol min^-^¹ mg^-^¹ protein), and **(D)** ascorbate (ASC) content (mg g^-^¹ FW). Significant differences (*p* < 0.05) are indicated by different letters according to Tukey’s HSD, while bars represent the standard error. Two-way ANOVA-based *p*-values for treatment, cultivar, and their interaction are shown in **(E)** with significant values in bold.

**Figure 8:**
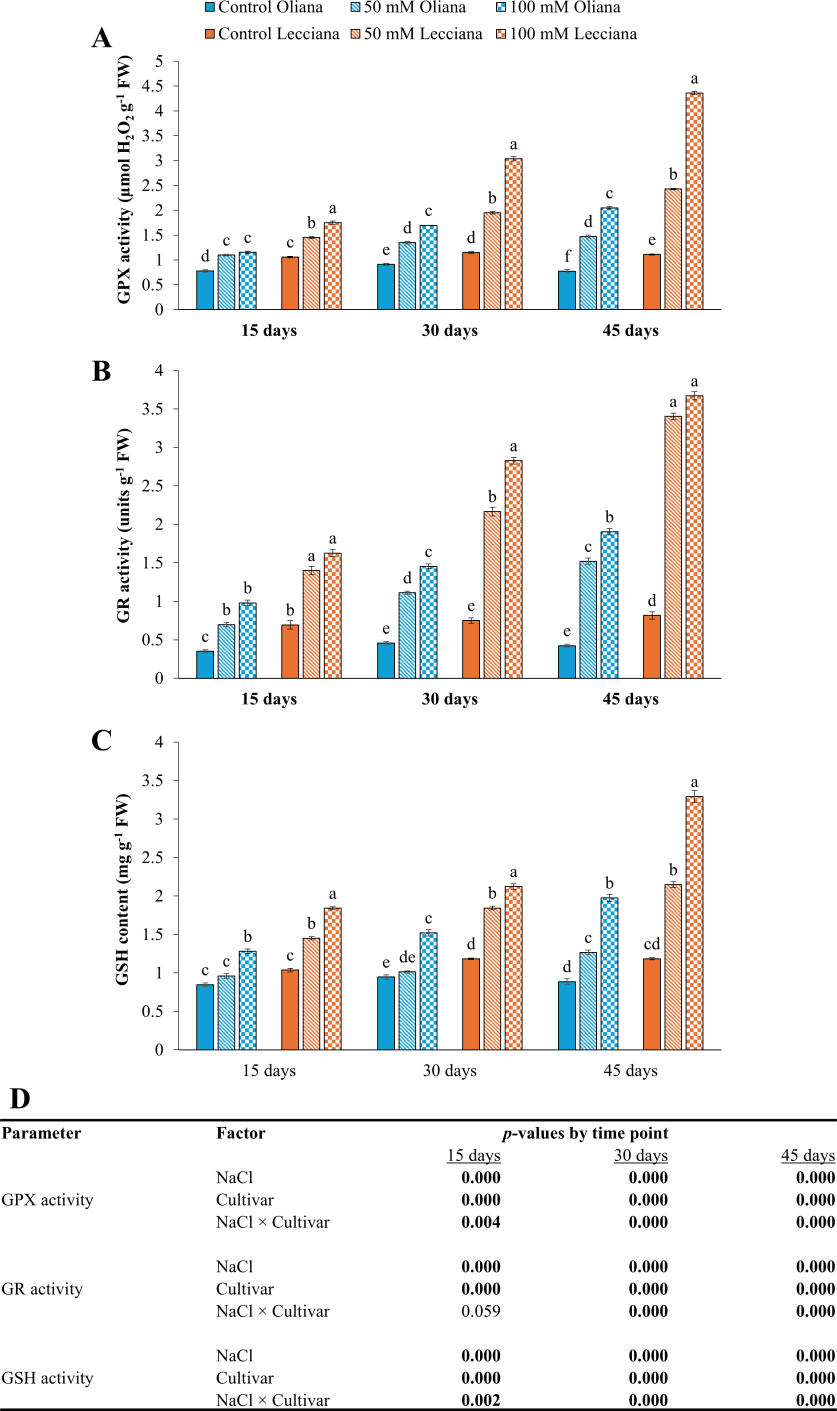
Impact of different salinity levels (0, 50, and 100 mM NaCl) on antioxidant enzyme activities of ‘Oliana’ and ‘Lecciana’ olive cultivars, measured at 15, 30, and 45 days after treatment (DAT). Measurements include **(A)** GPX activity (µmol H_2_O_2_ g^-^¹ FW), **(B)** GR activity (units g^-^¹ FW), and **(C)** GSH activity (mg g^-^¹ FW). Significant differences (*p* < 0.05) are indicated by different letters according to Tukey’s HSD, while bars represent the standard error. Two-way ANOVA-based *p*-values for treatment, cultivar, and their interaction are shown in **(D)** with significant values in bold.

Enzymatic activities including SOD, POD, CAT, APX, DHAR, MDAR, ASC, GPX, GR, and GSH were increased under NaCl treatments relative to the control and the ‘Lecciana’ cultivar showed the highest results at all the timepoints (15, 30, and 45 DAT) (**Figures 6**-**8**). Moreover, NaCl × Cultivar interaction was significant for most antioxidant enzymes, with SOD, APX, DHAR, ASC, GPX, and GSH, across all sampling times, whereas CAT, MDAR, and GR exhibited significant interactions mainly at later stages of stress (30 and 45 DAT), and POD showed interaction effects primarily at early to mid-stages (15 and 30 DAT) (**Figures 6A-D**, **7A-E**, **8A-D**).

### 3.6 Osmolyte concentrations of olive tree cultivars under salt stress

**Figure 9:**
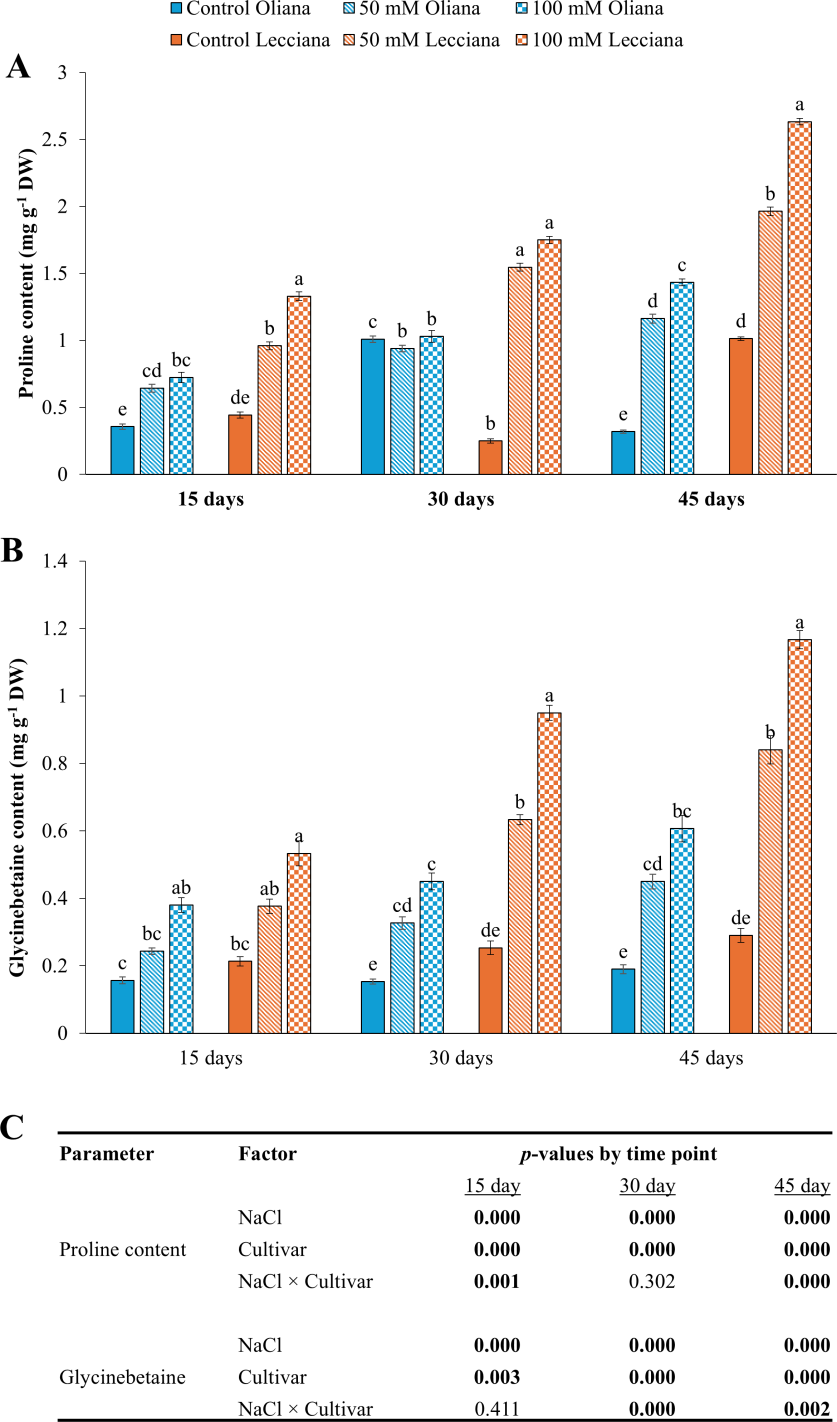
Impact of different salinity levels (0, 50, and 100 mM NaCl) on osmolyte accumulation in ‘Oliana’ and ‘Lecciana’ olive cultivars, measured at 15, 30, and 45 days after treatment (DAT). Measurements include **(A)** proline content (mg g^-^¹ DW) and **(B)** glycinebetaine (mg g^-^¹ DW). Significant differences (*p* < 0.05) are indicated by different letters according to Tukey’s HSD, while bars represent the standard error. Two-way ANOVA-based *p*-values for treatment, cultivar, and their interaction are shown in **(C)** with significant values in bold.

Osmolyte concentrations were increased under NaCl stress in both cultivars; however, highest increase was noted in the ‘Lecciana’ cultivar (**Figure 9**). The ANOVA results showed that NaCl treatments significantly affected the proline and GB contents at all timepoints (15, 30, and 45 DAT) and cultivars exhibited significant differences at all timepoints. However, the NaCl × Cultivar interaction was significant at 15 and 45 DAT for proline contents, while for glycinebetaine it was significant at 30 and 45 DAT (**Figures 9A-C**).

### 3.7 O_2_ content, H_2_O_2_ content, Lipid peroxidation, Electrolyte leakage of olive tree cultivars under salt stress

**Figure 10:**
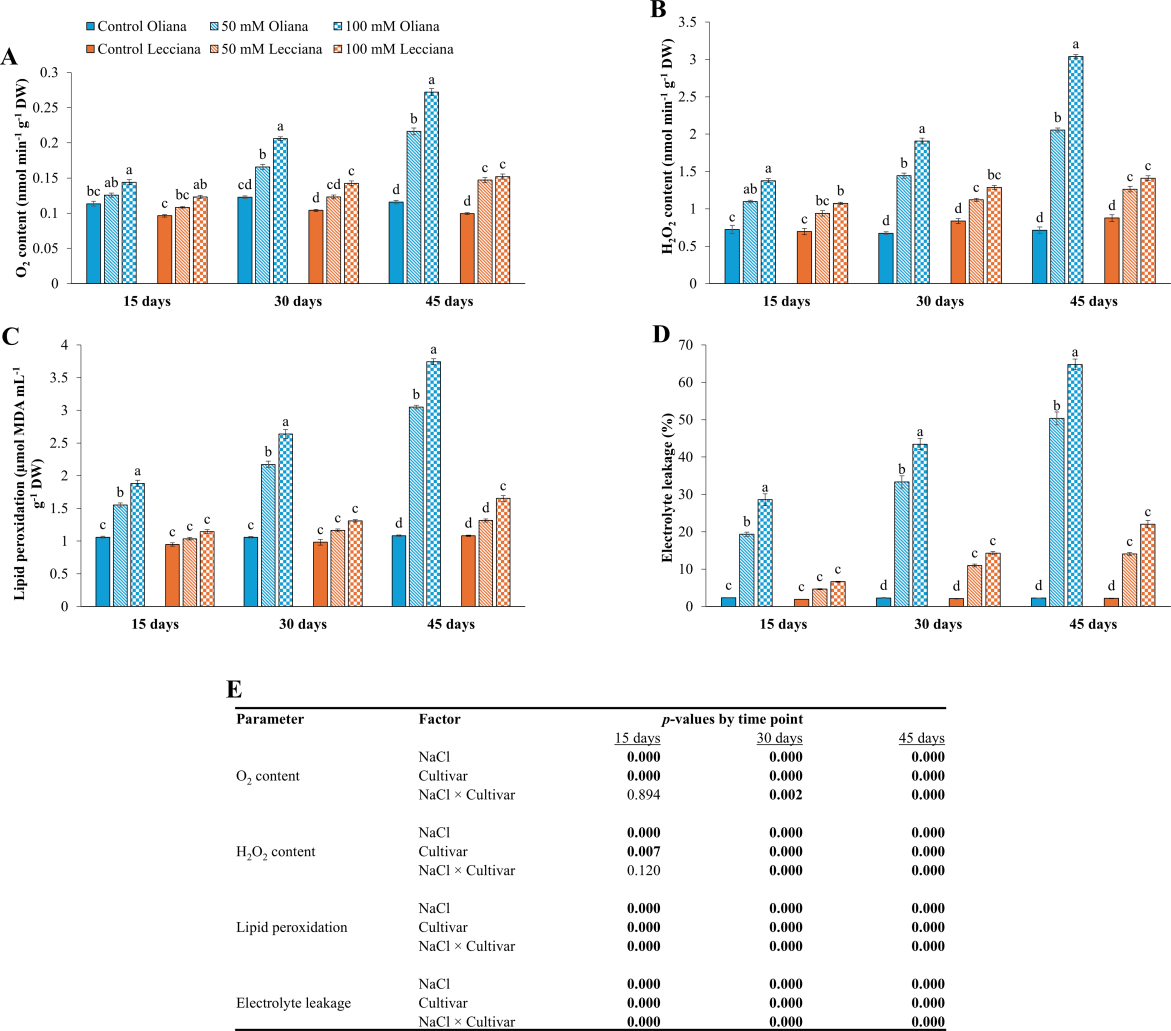
Impact of different salinity levels (0, 50, and 100 mM NaCl) on oxidative stress indicators in ‘Oliana’ and ‘Lecciana’ olive cultivars, measured at 15, 30, and 45 days after treatment (DAT). Measurements include **(A)** O_2_ content (nmol min^-^¹ g^-^¹ DW), **(B)** H_2_O_2_ content (nmol min^-^¹ g^-^¹ DW), **(C)** lipid peroxidation (µmol MDA mL^-^¹ g^-^¹ DW), and **(D)** electrolyte leakage (%). Significant differences (*p* < 0.05) are indicated by different letters according to Tukey’s HSD, while bars represent the standard error. Two-way ANOVA-based *p*-values for treatment, cultivar, and their interaction are shown in **(E)** with significant values in bold.

NaCl treatments elevated O_2_ and H_2_O_2_ contents, lipid peroxidation, and electrolyte leakage, particularly in the ‘Oliana’ cultivar (**Figure 10**). ANOVA results revealed that NaCl treatments impacted all these parameters, and significant differences were recorded between the cultivars at all the timepoints (15, 30, and 45 DAT). However, the interaction between NaCl treatments and cultivars was significant at 30 and 45 DAT for O_2_ and H_2_O_2_ contents while for lipid peroxidation and electrolyte leakage it was significant at all time-points (**Figures 10A-E**).

## 4 Discussion

Salinity stress generally impacts plant growth by causing water deficit, ionic imbalance and oxidative damage. Primary stresses generate ROS which leads to hormonal changes and affects the activities of certain enzymes (Munns, 2002). Antioxidants act as the primary defense mechanism against ROS (Mittler, 2017). In this study, enzymatic activities like SOD, POD, CAT, APX, DHAR, MDAR, ASC, GPX, GR, and GSH were increased under NaCl treatments as compared to the control treatment. Elevated antioxidants supports ROS regulation (Azooz et al., 2009), which minimizes the cellular injury and enhances the plant’s survival rate. The cultivar ‘Lecciana’ had higher antioxidant activities, which indicated an efficient ROS scavenging mechanism that translates to its high salinity tolerance. Higher antioxidative activities could contribute to maintaining photosynthetic efficiency (higher Fv/Fm), as well as minimized oxidative damage by reducing lipid peroxidation, which was indicated by lower MDA levels in ‘Lecciana’. The improved activities of APX, MDAR, and GR translates the activation of the AsA-GSH cycle, which facilitates efficient regeneration of reduced ascorbate and glutathione thereby strengthening the antioxidative defense within the cell. These results supports previous studies; for instance, Sofo et al. (2005) also demonstrated that in young olive plants of cultivar ‘Coratina’, CAT, APX and GPX activities were increased under water deficient conditions. Similarly, Regni et al. (2019) also reported that under salinity stress, four olive cultivars (‘Royal de Cazorla’, ‘Koroneiki’, ‘Fadak 86’, and ‘Arbequina’) demonstrated an increase in the GSH and CAT activity.

Additionally, osmolytes interact with the antioxidant enzymatic system to mitigate oxidative damage by reducing the overproduction of ROS, and maintaining the key antioxidant enzymes (i.e., SOD, CAT, APX) which prevent lipid peroxidation (Ejaz et al., 2020; Hasanuzzaman et al., 2021). The ‘Lecciana’ cultivar exhibited higher accumulation of osmolytes (proline and GB), indicating efficient osmotic adjustment potential and ROS detoxification ability compared to ‘Oliana’, which may contribute to its better adaptability to saline conditions in the current study. This enhanced antioxidative and osmotic adjustment mechanisms in cultivar ‘Lecciana’ minimized the oxidative and ionic stress which maintained ionic homeostasis by regulating Na^+^ and K^+^ balance across membranes. Consistently, Bashir et al. (2021) reported that more sustained accumulation of proline was recorded in the cultivar ‘Canino’ (salt tolerant) than the cultivar ‘Sirole’ (salt sensitive). In another study, Lu et al. (2021) reported that woody perennials i.e., *Tamarix ramosissima*, *Haloxylon ammodendron* showed enhanced tolerance to salinity induced oxidative stress through highly efficient and sustained antioxidant enzyme system to mitigate the oxidative damage. However, annual plants typically rely on rapid osmolyte, and antioxidant responses aimed at short-term stress avoidance and completion of their life cycle (Parvaneh et al., 2012; Cherifi et al., 2016; Alaei et al., 2020). Additionally, tolerant woody species may also reduce the toxic impacts of salinity stress through vacuolar compartmentalization of excess ions or their sequestration in bark, ray cells, tracheid walls and lumens, and older senescent leaves (Niknam and McComb, 2000).

In contrast to enhanced osmotic regulation and antioxidant defense in ‘Lecciana’ cultivar ‘Oliana’ demonstrated superior growth, gas exchange, and chlorophyll content which is likely attributed to different stress adaptation strategies between the two cultivars, as ‘Oliana’ may employ more ion exclusion or compartmentalization strategies. These salt avoidance mechanisms function by limiting salt uptake into the plant or by reducing cytoplasmic salt concentrations (Acosta-Motos et al., 2017). Additionally, this decreased salt uptake enhanced the nutrient uptake i.e., N, P, Ca, Mg, S, Zn, in leaves, stems, and roots of ‘Oliana’ cultivar which is likely due to its ability to maintain a better ionic balance. However, reduced growth and physiological responses and nutrient uptake in ‘Lecciana’ cultivar can be attributed to reduction in the cell division and elongation, which affects the transcriptional activity of major cell cycle regulators, i.e., cyclins and CDKs. This leads to reduction in meristematic cell numbers and overall plant growth (Balasubramaniam et al., 2023) which is consistent with the previous research on olive which indicated that moderate to high salinity levels adversely affect the growth and biomass (Chartzoulakis et al., 2002; Tabatabaei, 2006; Gholami et al., 2024). Similarly (Grattan and Grieve, 1998), reported that nutrient imbalance occurs due to competitive uptake and translocation under salinity stress which is consistent with the previous studies, that high salinity stress decreased the N, P, Ca, and Mg contents (Tattini et al., 1992; Klein et al., 1993; Tattini et al., 1995; El-Sayed Emtithal et al., 1996; Loupassaki et al., 2002; Kasırğa and Demiral, 2016).

**Figure 11:**
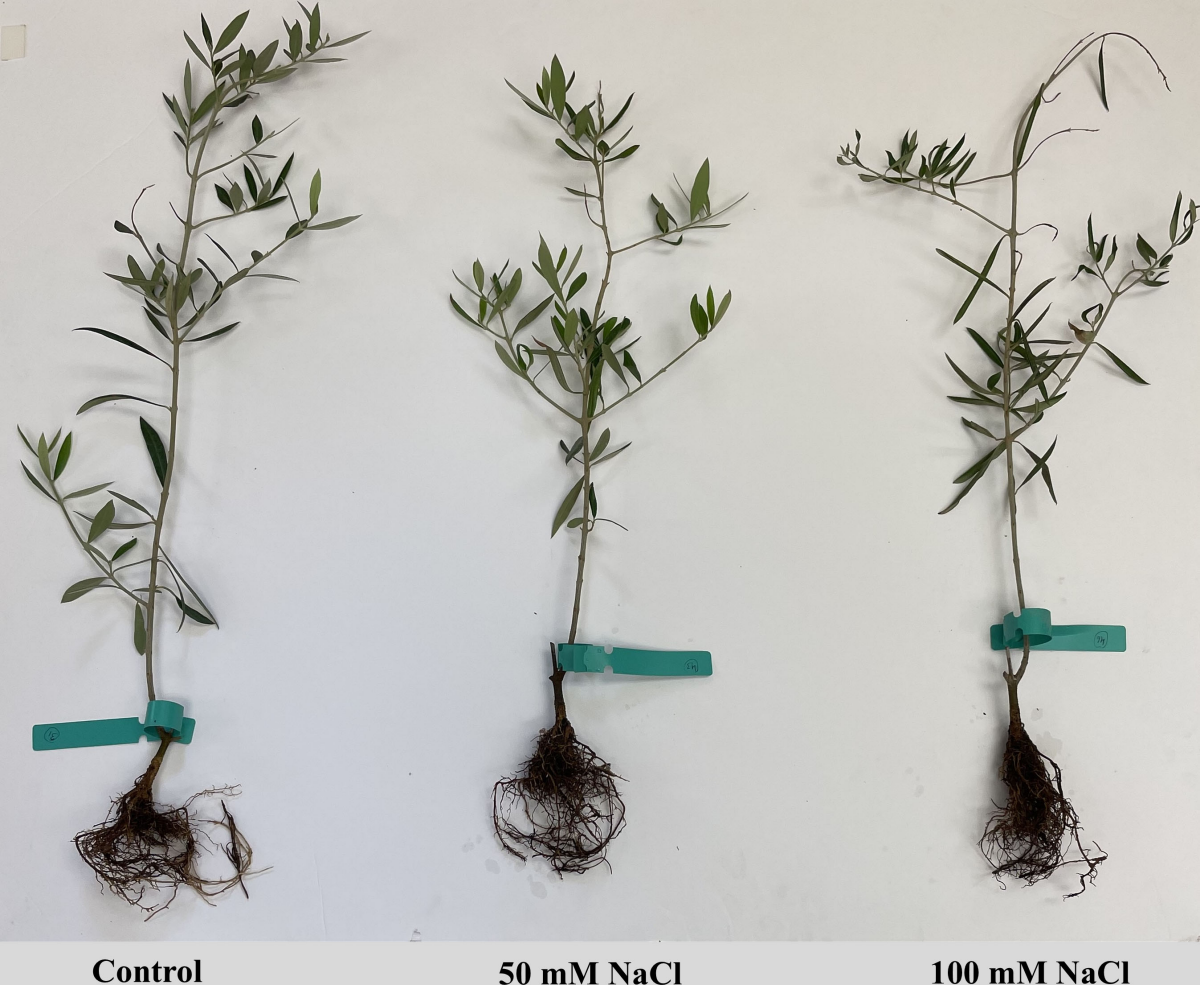
Representative image of three ‘Oliana’ plants illustrating visual differences at the end of the experiment under salinity treatments of 0 mM NaCl (control), 50 mM NaCl, and 100 mM NaCl.

**Figure 12:**
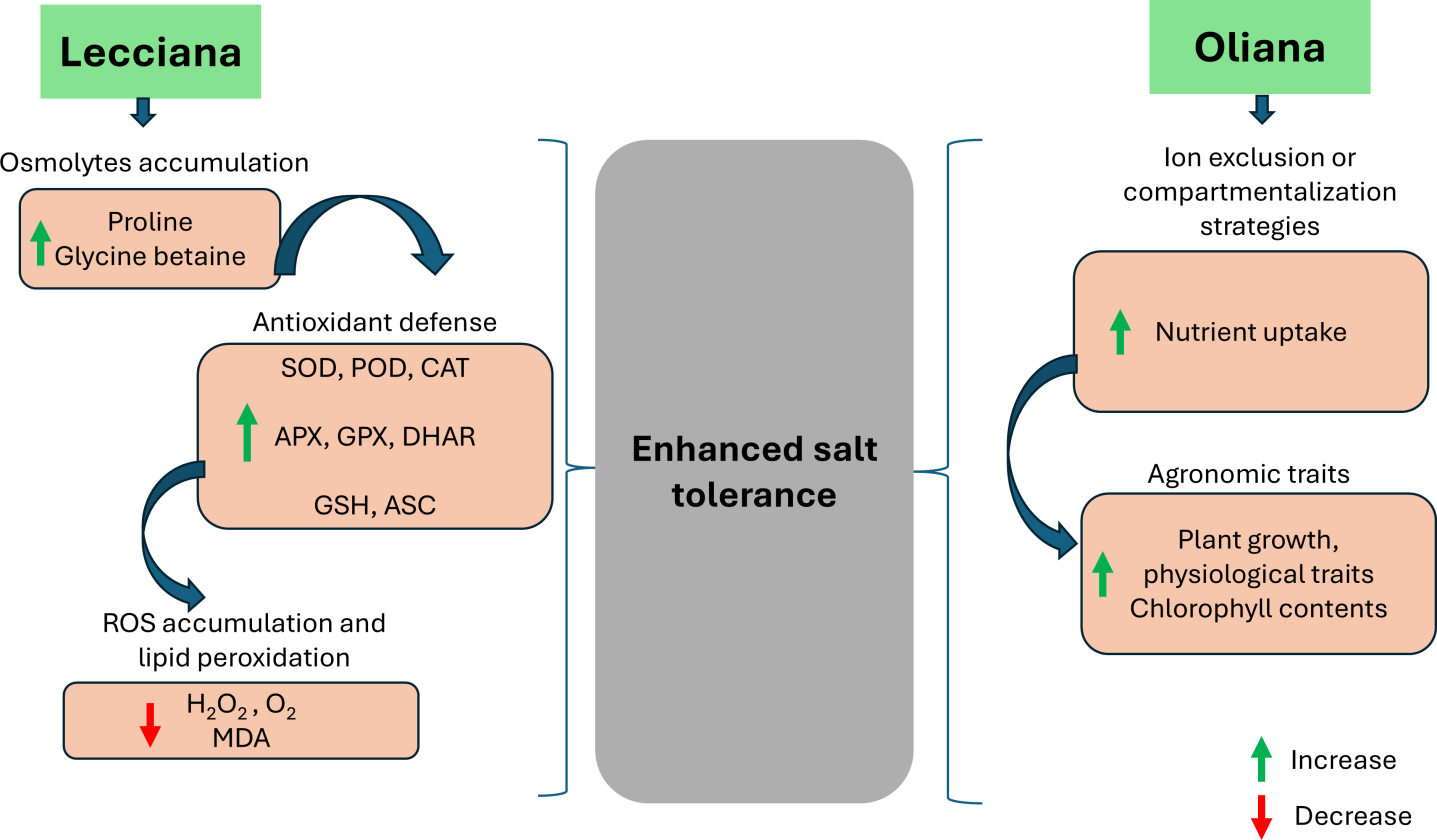
A graphical model representing the different response mechanisms of ‘Oliana’ and ‘Lecciana’ olive cultivars under salinity stress.

Overall, our findings showed that salinity stress activated an integrated antioxidant defense system in both olive cultivars, including both enzymatic (SOD, CAT, APX, GPX, GR) and non-enzymatic markers (AsA, GSH). However, the greater activation of these enzymes in ‘Lecciana’ strengthened its ROS scavenging ability and maintained chloroplast integrity and efficiency of photosynthetic machinery under salinity stress. The coordinated response explains the more efficient redox homeostasis in ‘Lecciana’ and its adaptation ability to saline conditions. In contrast, the ‘Oliana’ cultivar exhibited higher growth, physiological performance, and nutrient accumulation suggesting a stress avoidance strategy (**Figures 11**, **12**). The findings of this study reveal novel insights into cultivar dependent salt tolerance mechanisms in olive cultivars ‘Lecciana’ and ‘Oliana’ under greenhouse conditions. However, follow up field validation under natural saline conditions and investigation into molecular pathways associated with antioxidants biosynthesis and ROS detoxification to explore the mechanism of salt tolerance in depth is needed.

## Data Availability

The raw data supporting the conclusions of this article will be made available by the authors, without undue reservation.
